# A novel image augmentation based on statistical shape and intensity models: application to the segmentation of hip bones from CT images

**DOI:** 10.1186/s41747-023-00357-6

**Published:** 2023-08-08

**Authors:** Jérôme Schmid, Lazhari Assassi, Christophe Chênes

**Affiliations:** Geneva School of Health Sciences, HES-SO University of Applied Sciences and Arts Western Switzerland, Geneva, Switzerland

**Keywords:** Deep learning, Bone and bones, Hip joint, Tomography (x-ray, Computed), Total hip arthroplasty

## Abstract

**Background:**

The collection and annotation of medical images are hindered by data scarcity, privacy, and ethical reasons or limited resources, negatively affecting deep learning approaches. Data augmentation is often used to mitigate this problem, by generating synthetic images from training sets to improve the efficiency and generalization of deep learning models.

**Methods:**

We propose the novel use of statistical shape and intensity models (SSIM) to generate augmented images with variety in both shape and intensity of imaged structures and surroundings. The SSIM uses segmentations from training images to create co-registered tetrahedral meshes of the structures and to efficiently encode image intensity in their interior with Bernstein polynomials. In the context of segmentation of hip joint (pathological) bones from retrospective computed tomography images of 232 patients, we compared the impact of SSIM-based and basic augmentations on the performance of a U-Net model.

**Results:**

In a fivefold cross-validation, the SSIM augmentation improved segmentation robustness and accuracy. In particular, the combination of basic and SSIM augmentation outperformed trained models not using any augmentation, or relying exclusively on a simple form of augmentation, achieving Dice similarity coefficient and Hausdorff distance of 0.95 [0.93–0.96] and 6.16 [4.90–8.08] mm (median [25th–75th percentiles]), comparable to previous work on pathological hip segmentation.

**Conclusions:**

We proposed a novel augmentation varying both the shape and appearance of structures in generated images. Tested on bone segmentation, our approach is generalizable to other structures or tasks such as classification, as long as SSIM can be built from training data.

**Relevance statement:**

Our data augmentation approach produces realistic shape and appearance variations of structures in generated images, which supports the clinical adoption of AI in radiology by alleviating the collection of clinical imaging data and by improving the performance of AI applications.

**Key points:**

• Data augmentation generally improves the accuracy and generalization of deep learning models.

• Traditional data augmentation does not consider the appearance of imaged structures.

• Statistical shape and intensity models (SSIM) synthetically generate variations of imaged structures.

• SSIM support novel augmentation approaches, demonstrated with computed tomography bone segmentation.

**Graphical abstract:**

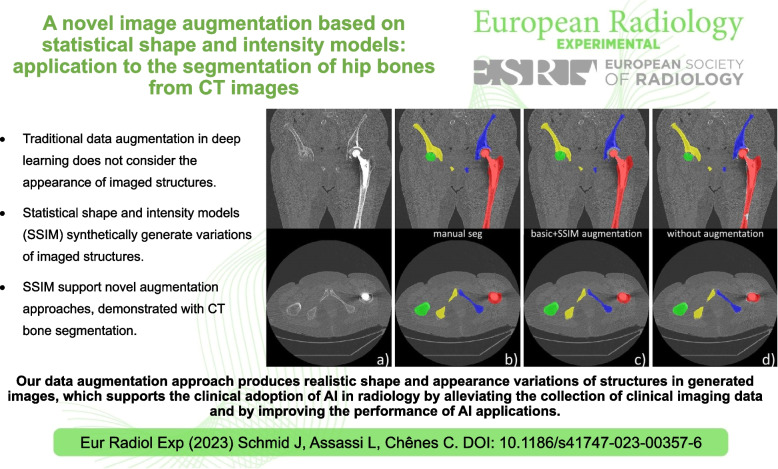

## Background

Like in many fields, deep learning has enabled major advances in radiology, thanks to modern computing capabilities and access to big data. Data is particularly critical for deep learning performance. In medical imaging, access to data is hindered by several factors such as low prevalence of pathologies, effort required for data annotation, patient privacy, and ethical concerns [1], representing one of the main obstacles to the application of efficient deep learning-based algorithms. To overcome the limited number of images available, several strategies have been investigated such as shallower neural networks (often with loss of performance), transfer and zero-shot learning, or data augmentation [1–4]. Data augmentation focuses on the root of the data problem and aims at increasing the size and diversity of the training set by synthetically creating new data samples. Data augmentation is often seen as a type of regularization to improve the generalization of deep learning models [5] by avoiding overfitting and countering data imbalance [3]. While several works are still investigating the complex theoretical foundations of data augmentation [6, 7], there is a general consensus that data augmentation is beneficial, especially with small datasets [1].

Many different taxonomies of data augmentation have been proposed [1–4]; we can mainly classify augmentation techniques in terms of complexity (basic *versus* advanced approaches) or targeted image domains (geometrical or photometric). For instance, basic approaches include geometrical transformations (rotations, flipping, etc.) or intensity modification (noise injection, edge enhancement, smoothing, etc.). Advanced approaches include deep learning approaches, especially based on generative adversarial networks (GAN) [8], as well as approaches based on statistical models [9–12]. GAN-based data augmentation creates samples with variations in both shape and intensity of generated structures, while augmentation using statistical models will alter the geometry of existing images. In fact, these models mainly rely on statistical shape models (SSM), where generated instances by the SSM provide spatial deformations [9, 10] or mesh representations [11, 12] that can be used to geometrically deforms a training image — resulting in an augmented image. While this concept can generate many new training images, the resulting image intensities are restricted by the initial training images.

As a result, we propose in this work to enhance SSM-based augmentation by investigating the use of a statistical shape and intensity model (SSIM) modeling both shape and intensities of imaged structures. To our best knowledge, no previous work has exploited statistical intensity models (SIM) to directly generate augmented samples, despite the long existence of SIM in medical image analysis, often under the name of appearance models[13].^1^ The closest work [14] created an active contour model encompassing shape contours with intensity information to guide an image-to-image conditional GAN for data augmentation, in the context of cell segmentation from optics retinal imaging.

Assessing the efficiency and impact of a new data augmentation technique is usually done in the context of a specific application. In this work, we chose the task of segmenting bones from computed tomography (CT) images for total hip arthroplasty (THA) planning. Most works that relied on deep learning to segment (some) bones of the hip from CT in the context of THA [15, 16] used basic geometric augmentations such as rotation, translation, scaling, cropping, and left–right flipping. This was also observed with other studies that did not focus on THA but also segmented bones of the hip joint from CT [17] and sometimes included simple intensity augmentations such as intensity scaling [18]. Except for the work of Noguchi et al. [19] that exploited more advanced augmentation (*e.g.*, mix-up and patching) for whole-body bone segmentation in CT, most previous works were thus based on simple augmentation techniques. As a result, our more advanced augmentation approach brings further novelty with respect to these previous works.

## Methods

### Overview of the augmentation pipeline

**Fig. 1:**
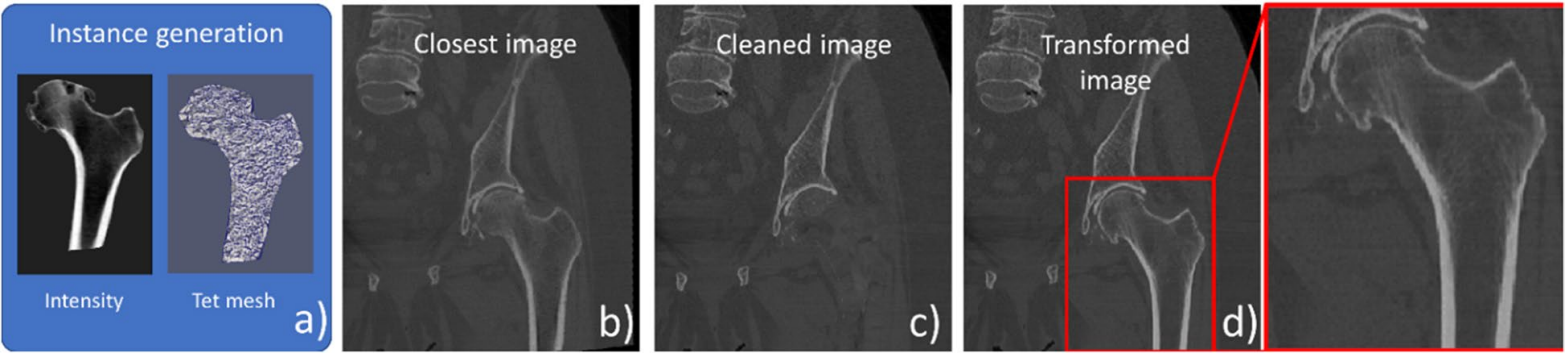
The main steps of a new computed tomography image generation exemplified with the left proximal femur. Random sampling of instance using a statistical shape and intensity model (SSIM) (**a**). Selection of closest image based on shape affine similarity (**b**). Cleaning of corresponding structure in the closest image (**c**). Geometrical transform of cleaned image with painting of instance intensities (**d**)

The aim of the augmentation method is to generate diversified and realistic images to train a machine learning approach. In standard augmentation for supervised learning, we generate new image samples from a collection of training images with corresponding labels, *i.e.*, in our application segmentation masks for our four bones (left/right proximal femurs and hip bones). For a given structure, a new image is created as follows (Fig. 1):

- Constrained random sampling of a tetrahedral mesh with embedded image intensities using the SSIM (Fig. 1a)
- Selection of the closest image in the training set based on an affine distance criterion between the sampled mesh and the corresponding mesh of the closest image (Fig. 1b)
- Cleaning of the closest image by “removing” the intensities of the corresponding bone (Fig. 1c)
- Warping of the cleaned image using thin-plate spline (TPS) and rigid transformations along with the “painting” of the sampled synthetic image intensities (Fig. 1d)

For the sake of clarity, we assume in the following sections that we have a single structure to segment the overall approach being simply replicated for other structures.

### Instance generation based on SSIM

Given a series of images with the segmented structure, we non-rigidly registered a reference triangular mesh to the segmented structure of each training image — establishing a point correspondence between the registered meshes. The reference model was then converted to a volumetric tetrahedral mesh [20] that was warped to each image space using a thin-plate spline transform (computed with a subset of the mesh surface vertices). Given co-registered tetrahedral meshes with a point correspondence of their vertices, we eventually built a statistical shape model with rigid Procrustes alignment, modeled as a point distribution model. To express image intensities in the interior of meshes, we exploited the compact representation of CT intensities within a tetrahedron as a continuous density function, proposed by Yao [21]. Using Bernstein polynomials, the density function models within a tetrahedron the image intensity $D\left(p\right)$ at a position ${p={( p}_{x}{,p}_{y} {,p}_{z}, p}_{w})$ defined in a local barycentric coordinates space:

$$D\left(p\right)={\sum }_{i+j+k+l=d}\left({C}_{i,j,k,l}.{B}_{i,j,k,l}^{d}(p)\right)$$

where ${B}_{i,j,k,l}^{d}$ is the barycentric Bernstein function of degree $d$ and ${C}_{i,j,k,l}$ are the Bernstein coefficients, computed by solving a system of equations. The larger the degree $d$ is, the better will be the fidelity of the encoded intensities but at the expense of large memory/storage requirements — since the number of Bernstein coefficients $m$ per tetrahedron quickly increases ($m=\frac{\left(d+3\right)!}{3!d!}$). Similarly to SSM that use vertex positions to build their point distribution model using principal component analysis (PCA), the SIM will use the Bernstein coefficients.

**Fig. 2:**
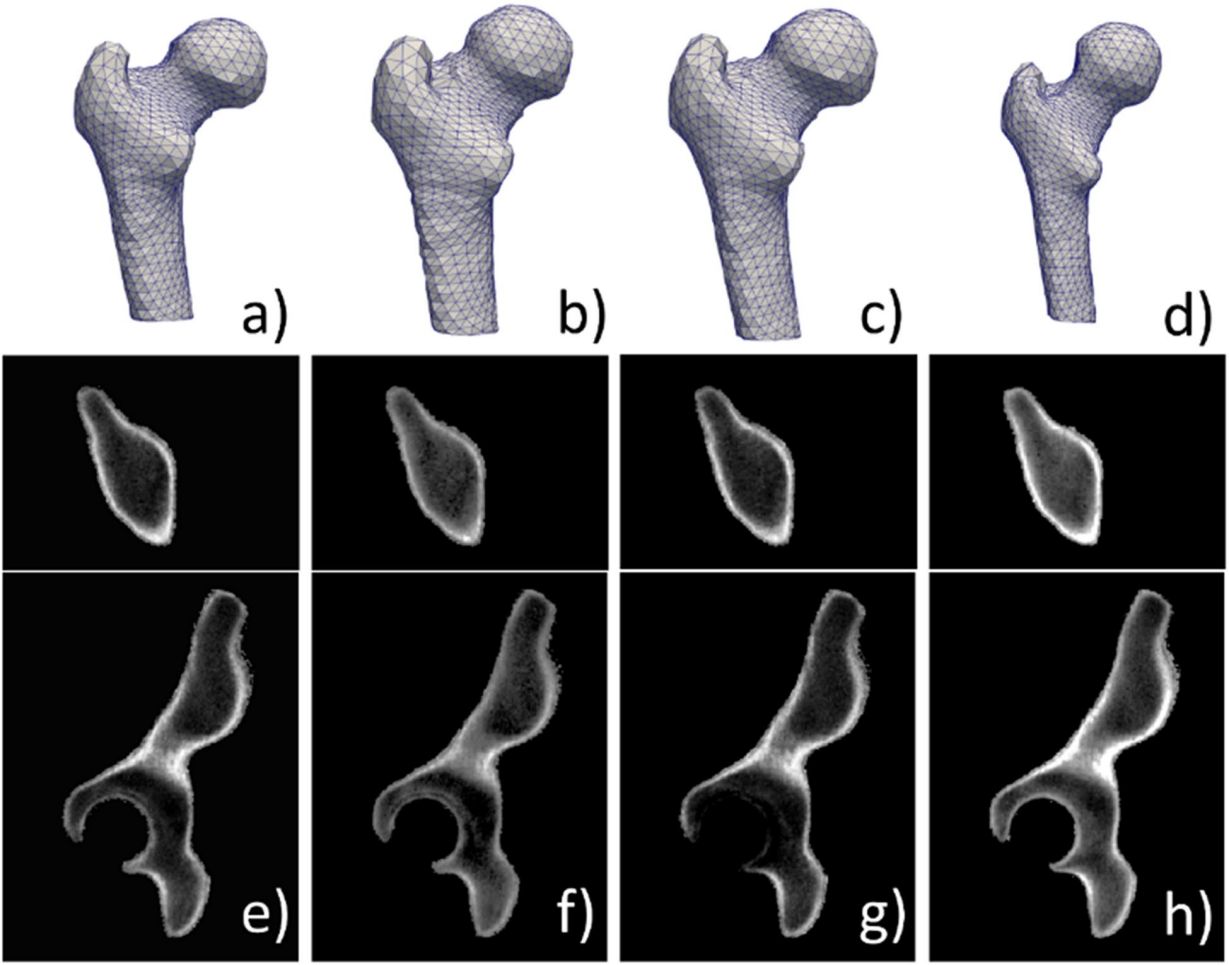
Example of generated instances using a statistical shape and intensity model (SSIM). The statistical shape model produces different tetrahedral meshes of a proximal femur (**a**–**d**). The statistical intensity model yields different intensity appearance for the same hipbone tetrahedral mesh: top, axial view; bottom, sagittal view (**e**–**h**)

By unifying the two statistical models into a SSIM, we can generate shape and intensity instances by varying the model statistical parameters ${b}_{i}$, as depicted in Fig. 2. Parameters ${b}_{i}$ follow a multivariate Gaussian distribution with variances equal to the eigenvalues ${\lambda }_{i}$ of the PCA. As a result, a random generation of a new instance is easily achieved by sampling the multivariate Gaussian distribution [12]. However, special care must be taken in constraining this sampling to avoid extreme unrealistic shape or intensities. Similarly to [11, 12], we restrict the ${b}_{i}$ to an interval $\left[-2.5\sqrt{{\lambda }_{i}},2.5\sqrt{{\lambda }_{i}}\right]$. We further scale the parameters so that $\sum \frac{{b}_{i}^{2}}{{\lambda }_{i}}\le M$, where $M$ derives from the ${\chi }_{2}$ distribution [22].

### Creation of augmented images

Given a shape-intensity instance generated by the SSIM, we find the image whose segmented structure is the closest (in terms of Euclidean distance [11, 12]) to the shape of the instance after affine registration. Then we proceed to “clean” the structure in the closest image by erasing it (*e.g.*, Fig. 1c). Using the segmentation mask, we compute a signed distance map which allows us to find for any voxel $v$ in the interior of the structure the point $w$ on the segmentation border that is the closest. Then, we replace the intensity of $v$ by the intensity of the exterior point $r$ at $v+2 \left(w-v\right)$, similarly to a mirror-like approach in image border extrapolation.

Using the TPS transforming the instance to the closest segmented structure, we resample the cleaned image into the instance coordinate system as performed in previous works [9–12]. This produces some slight deformations of surrounding structures, achieving a first degree of augmentation. We go further by painting the intensities of the instance in the resampled image. Finally, we rigidly align the resampled image with the closest image to reduce some overall rigid motions caused by computing the TPS with small or not centered structure such as a femur.

The reason of doing the cleaning is to reduce the risk that some intensities of the original structure remain after painting of the instance intensities due to small errors of the TPS warping, especially in the vicinity of its border, causing some kind of “ghosting” effect.

### Application of augmentation for CT bone segmentation

We evaluated the impact of our augmentation technique in the context of hip joint bone segmentation from THA preoperative CT images, which often presented pathological structures. A partner provided a retrospective anonymized dataset including 232 patients (112 men and 120 women, with average and median age of 74.4 and 75.4 years) gathered from various clinical institutions. For each patient, were included a preoperative THA CT image with corresponding manual segmentation of hip bones and proximal femurs. Using a Likert scale from 1 (low) to 3 (high), three radiographers analyzed the images and rated the overall image quality and noise level at 1.7 and 0.75, respectively. They also spotted image artifacts in 25% of the images, mostly metallic artifacts commonly caused by the presence of previous hip implants (around 23% of patients). Osteophytes were also observed on more than 65% of the patients’ hips to be operated.

Using the CT images and the segmentations, we trained a residual U-Net architecture [23] using a fivefold cross-validation. Given a fold, 4/5 of the patients were randomly chosen for training data, while the remaining images were used for testing. Within the training set, 10% of patients were reserved for the validation set. For each fold, we built a SSIM model with the fold training data. Based on the series of patient images in the training and validation datasets, different augmentation techniques were applied offline or on-the-fly to generate new images (*i.e.*, new “samples”):

- “No-aug”: No augmentation was used, the number of samples was hence equal to the number of patients, in the training and validation datasets.
- “Basic”: A basic augmentation was used based on standard intensity and geometrical transformations, which were randomly applied on-the-fly during the training with a probability $p$: vertical and horizontal flipping ($p$ = 0.1), 90° rotation ($p$ = 0.1), and intensity shifting of ± 10% ($p$ = 0.5).
- “SSIM”: Our SSIM-based augmentation was used offline to augment the training and validation datasets.
- “Basic + SSIM”: The SSIM-based augmented samples were also augmented on-the-fly with the basic augmentation approach.

**Fig. 3:**
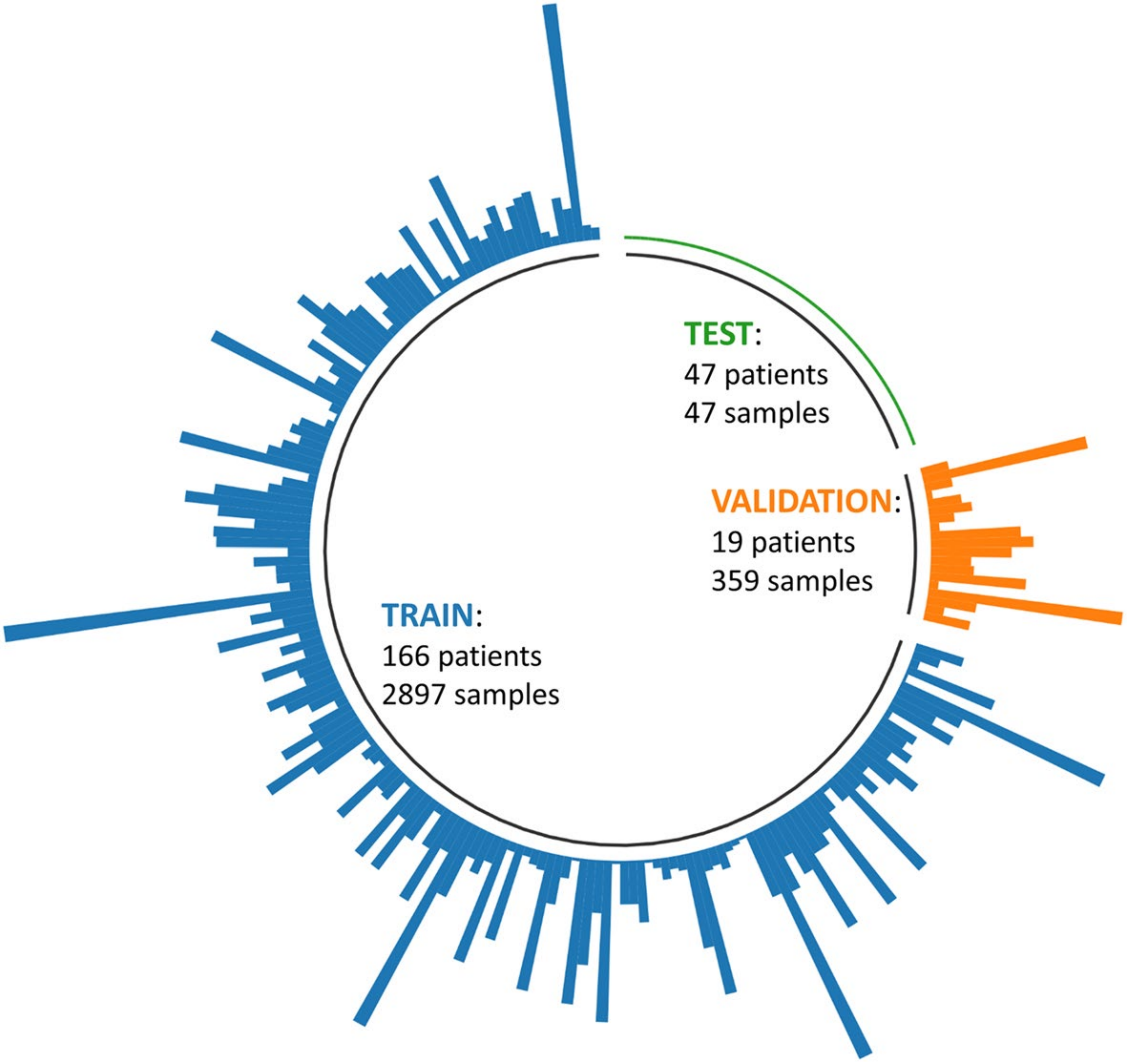
Distribution of patients and generated samples by augmentation for fold 0 and each type of dataset

For the SSIM-based augmentations, we tried to balance the use of patients; otherwise, some patient images would have been never chosen based on the closest image criterion, but we did not enforce perfect balance as the criterion is necessary to prevent excessive nonrealistic deformations caused by the TPS warping. For example, Fig. 3 depicts the resulting distribution for fold 0. Since for each patient we could augment based on each type of bone, this brought additional variety to the augmented samples.

We relied on the Dice similarity coefficient (DSC) and the Hausdorff distance (HD) to assess segmentation results against manual reference segmentation performed by trained radiographers. By using the results of the folds testing, approaches were compared with two-sided Mann–Whitney-Wilcoxon tests with Bonferroni correction [24] to account for multiple comparisons with a significance level at 0.05. The choice of the nonparametric test was motivated by the non-normality of the data, verified with a Shapiro–Wilk test and visual assessment of corresponding *Q*-*Q* plots.

### Implementation details

Our implementation relied on MONAI library [25] that is built on top of the PyTorch deep learning framework [26]. Our hardware was 4 × NVIDIA Tesla V100 SXM2 with 32 GB of GPU RAM. During training in addition to possible subsequent on-the-fly augmentation, all loaded images were initially normalized by a spatial resampling to an isotropic 1.5 mm^3^ voxel size and a rescaling of image intensities to the interval [0, 1]. We relied on a smart cache mechanism of MONAI that exploits images (transformed for normalization or augmentation) located on GPU caches to keep GPUs busy at each epoch: 80 images were initially put in each GPU cache, and at each epoch, 75% of the images were replaced, for a distributed training over 50 epochs. The final model was the one producing the best DICE coefficient with the validation dataset, which was tested every 30 images. We relied on MONAI library’s implementation of residual U-Net using default parameters with single-channel images. We used a loss summing the contributions of the DICE metric and cross-entropy, along with an Adam optimizer with a learning rate of 0.0001.

Ensemble models were built using the trained model of each fold. When tested on a non-segmented image, the segmentation masks inferred by each fold model were then averaged with weights corresponding to the average DICE score of each model when tested on their test datasets (obtained during the cross-validation experiments).

## Results

### Statistical shape and intensity models

On average, proximal femur and hipbone tetrahedral meshes had 8,734/52,220 and 20,626/116,062 vertices/tetrahedra, respectively. Statistical shape and intensity models were both built by keeping 95% of the total variance, which resulted in different numbers of modes for the proximal femur (17 and 71 modes for SSM and SIM) and hipbone (50 and 151 modes for SSM and SIM).

**Fig. 4:**
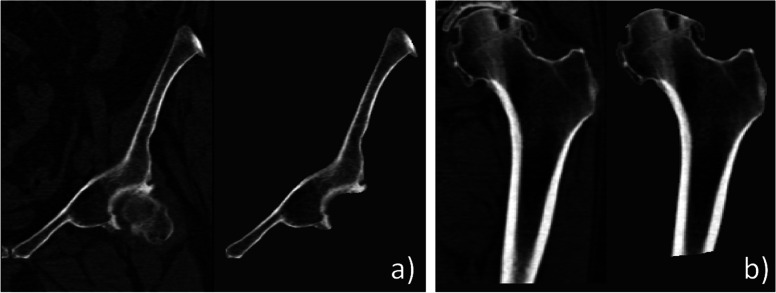
Example of reconstructed intensities after encoding with Bernstein polynomials of degree 3 for a hipbone (**a**) and proximal femur (**b**). For each bone, the left subfigure corresponds to the computed tomography image, while the right is the reconstructed image using the encoded intensities

When building the SIM, the degree $d$ of Bernstein polynomials we decided to set to 3, resulting in $m=20$ Bernstein coefficients per tetrahedron. In fact, given the high number of coefficients and tetrahedra, resulting models are usually very large compared to SSM, so we had to balance the resolution of the mesh and the degree $d$ to find a good compromise in terms of intensity fidelity and memory/storage constraints. To assess the impact of this choice of degree, we measured the absolute relative error in percentage between the CT intensities and those obtained by using the encoding with Bernstein polynomials. An error below 10% was obtained, with an excellent visual fidelity as depicted in Fig. 4.

### Segmentation results

**Fig. 5:**
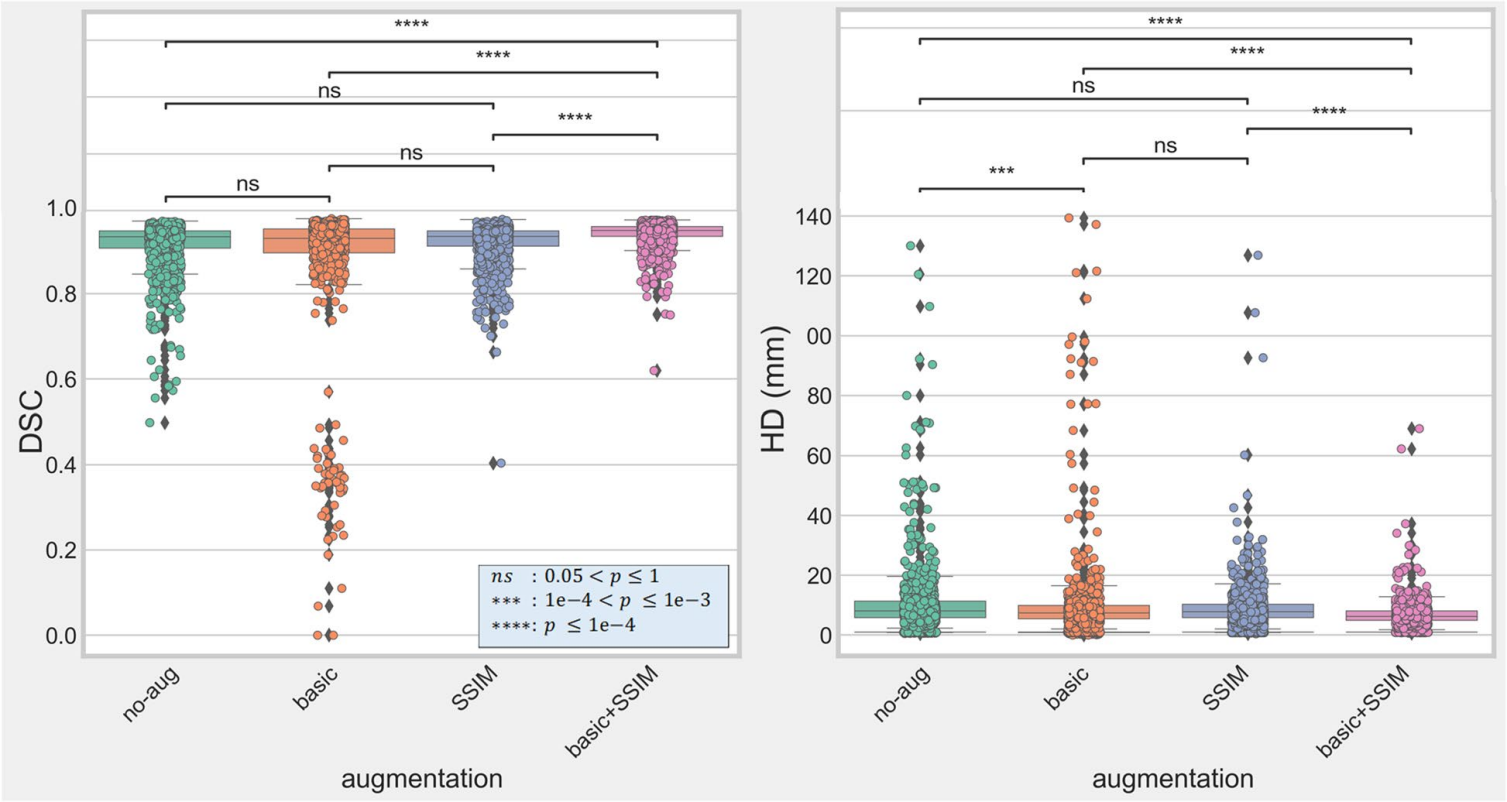
Box plots of the fivefold cross-validation results for the four augmentation methods. Approaches not using the statistical shape and intensity model (SSIM) augmentation generally present a high number of outliers (black diamonds). Statistical significance between methods is reported with “*” and “ns” standing for the absence of significance

Segmentation results are reported in Table 1 and Fig. 5, considering the data of all folds and for the 4 bones. In general, the two augmentation approaches using SSIM yielded better descriptive statistics (Table 1), although statistical significance on both DSC and HD metrics was only observed with the fully augmented approach Basic + SSIM (Fig. 5). Despite the absence of statistical significance for the SSIM augmentation alone, Fig. 5 highlights that many outliers occurred with the other two approaches, especially the basic augmentation with the DSC measure, which depict some large global segmentation errors. As a consequence, we also report in Table 1 the median and interquartile interval to better appreciate the performance differences between methods. The use of the basic augmentation was positive in terms of HD for both the segmentation models trained on original data (median no-aug: 8.6 *versus* basic: 7.35 mm, $p$ = 0.0004 or pre-augmented data with SSIM (median Basic + SSIM: 6.16 *versus* SSIM: 7.81 mm, $p$-value < 0.0001). For the DSC measure, no statistical difference could be proven between the basic and no-aug.

**Table 1:** Segmentation results reported as mean ± standard deviation and median (25th−75th percentiles) for the fivefold cross-validation using the different augmentation approaches

	No augmentation	Basic	SSIM	Basic + SSIM
DSC (mean ± standard deviation)	0.91 ± 0.06	0.89 ± 0.14	0.92 ± 0.05	0.94 ± 0.03
DSC (median [25th–75th percentile])	0.93 [0.90–0.95]	0.93 [0.90–0.95]	0.93 [0.91–0.95]	0.95 [0.93–0.96]
HD (mm) (mean ± standard deviation)	10.8 ± 11.5	9.9 ± 13.3	9.1 ± 7.7	7.0 ± 4.5
HD (mm) (median [25th–75th percentile])	8.06 [5.83–11.36]	7.35 [5.48–9.90]	7.81 [5.83–10.36]	6.16 [4.90–8.08]

*Basic* Basic augmentation, *Basic* + *SSIM* Basic augmentation coupled with augmentation using statistical shape and intensity model, *DSC* Dice similarity coefficient, *HD* Hausdorff distance, *SSIM* Augmentation using statistical shape and intensity model

## Discussion

**Fig. 6:**
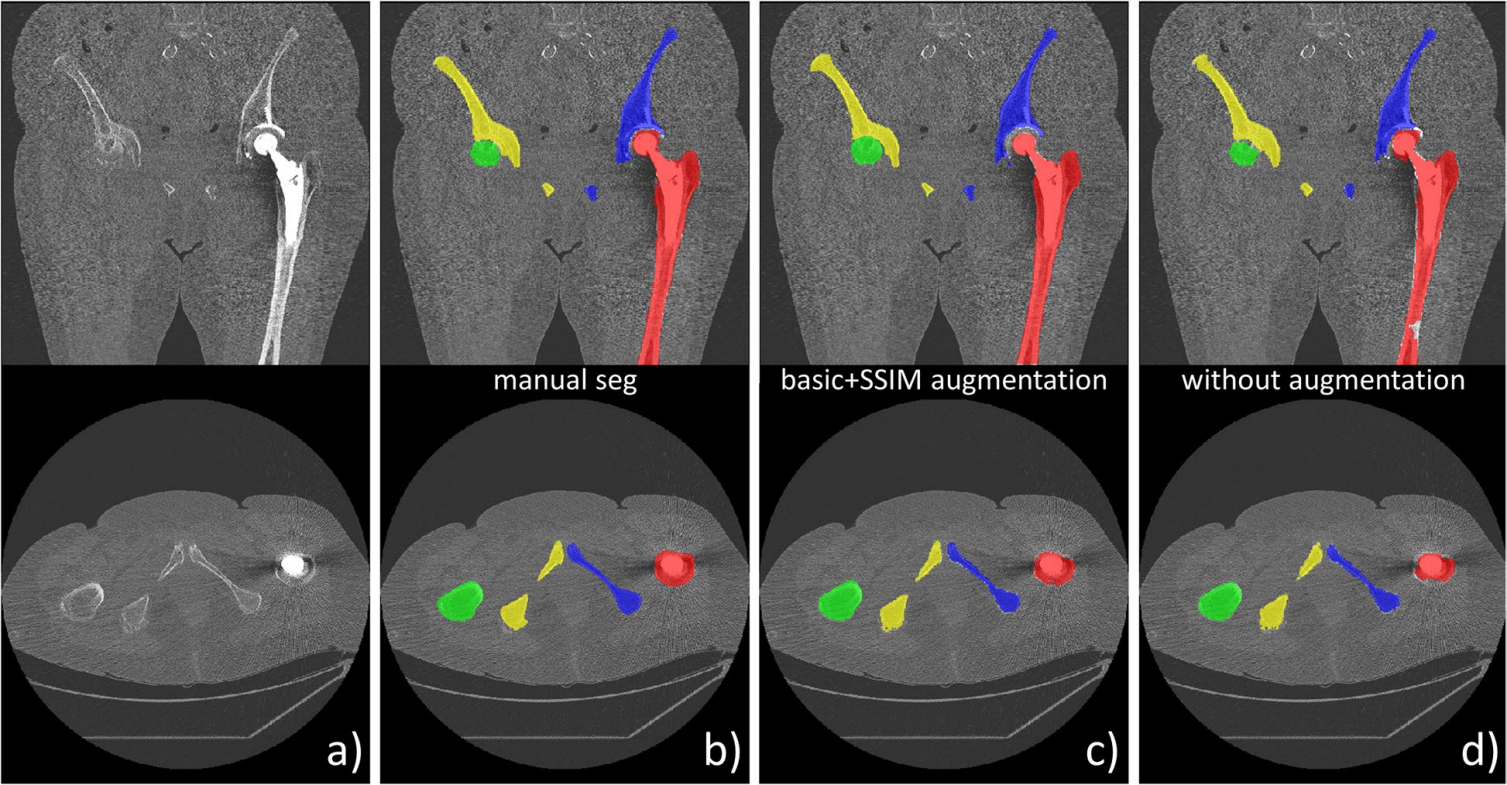
Comparison of segmentation results for a noisy computed tomography image with an implant in the left femur (**a**). Compared to the manual segmentation (**b**), the Basic + SSIM augmentation strategy (**c**) produced better results (*DSC* = 0.93, *HD* = 7.05 mm) than a network trained without any augmentation (**d**) (*DSC* = 0.88, *HD* = 12.4 mm)

According to Chlap et al. classification of medical imaging augmentation techniques [3], our approach could be categorized as a deformable augmentation for its image deformation capabilities but also as a kind of intensity generative model more commonly offered by augmentation approaches using GAN. As reported by several works [1–4], we found that augmentation was in general beneficial. Indeed, the absence of any augmentation resulted in a significant decrease of performance compared to the best augmentation approach “Basic + SSIM” as confirmed by the metrics (median DSC/HD: 0.93/8.06 *versus* 0.95/6.16) and as illustrated in Fig. 6 with a noisy CT image including a metallic implant. Furthermore, the accurate augmentation “Basic + SSIM” approach was also the most robust as it resulted in less outliers, as shown in Fig. 5 and confirmed by smaller differences between median/mean and standard deviation/interquartile ranges (equal to 75th–25th percentiles). The Basic + SSIM augmentation also outperformed the basic augmentation, highlighting the benefits of SSIM able to alter both spatial and intensity information. The use of basic augmentation was in appearance beneficial when applied to the original dataset but only in terms of HD which can be the consequence of highly localized large segmentation errors. However, the basic augmentation combined with our SSIM augmentation did boost the performances, showing that basic transformations can still provide some additional variety to improve segmentation robustness and accuracy.

**Fig. 7:**
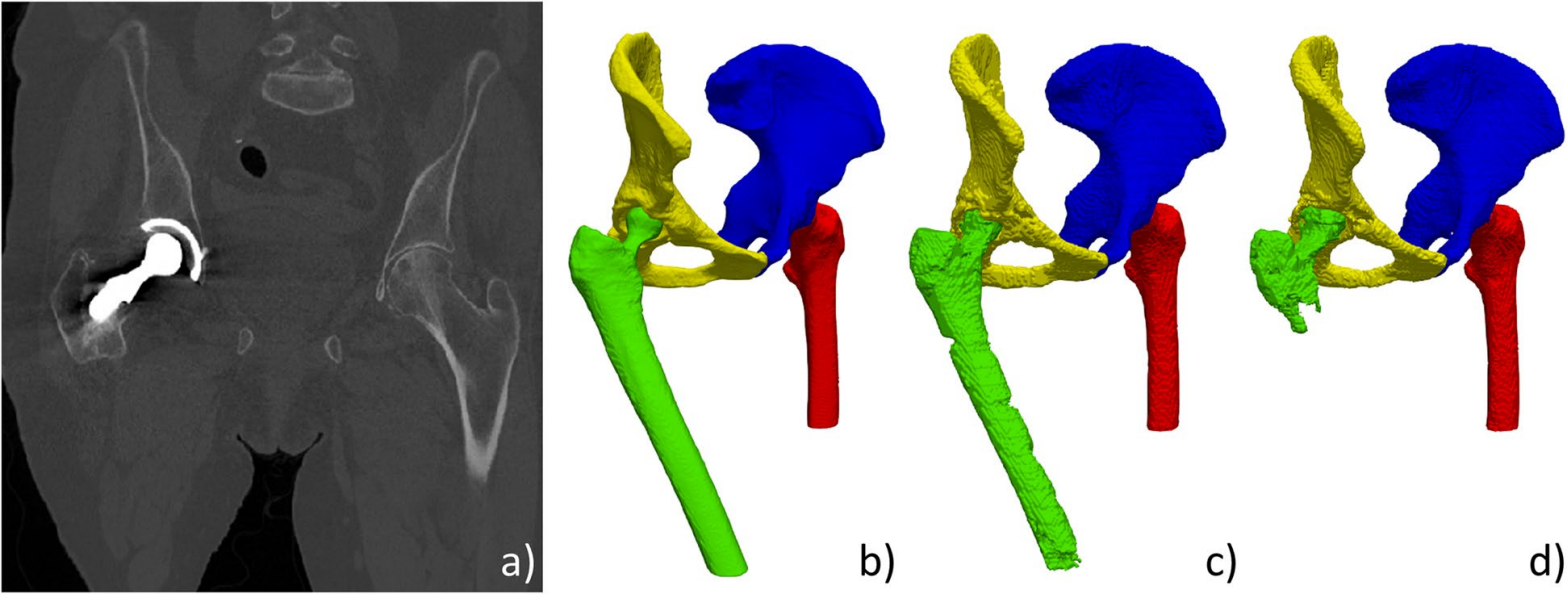
Example where the statistical shape and intensity model (SSIM) augmentation was not sufficient. Computed tomography image with implant in the right femur and nonconventional leg position (**a**) as depicted in the reference manual segmentation (**b**). Despite not being excellent, the right femur segmentation with the Basic + SSIM augmentation (**b**) is clearly superior to the segmentation result when only SSIM augmentation was used for training (**c**)

This is exemplified in Fig. 7 where a model trained with an augmentation only using SSIM could not cope with a femur unconventionally oriented and presenting an implant causing metallic artifacts.

We did not attempt to optimize the segmentation approach (type of model architecture, training parameters, etc.) as we focused on the design of a new augmentation approach. As long as statistical shape models can be built, using, *e.g.*, (semi-)automatic approaches such as those described in the literature [12, 27], the other major steps (tetrahedralization, intensity encoding with Bernstein polynomials, etc.) can be applied, resulting in a generalizable augmentation approach. In general, for any body part, with possibly more complex (varying) anatomy compared to the hip, the most challenging part remains the creation of the statistical shape models. Thankfully, this area has been investigated in the past, with several previous works reporting successful statistical models for complex anatomies such as the (growing) maxillofacial region [28, 29] or the spine [30, 31].

As our dataset is being composed of acquisitions from several institutions with different patient population and scanners, we found that our best model (DSC 0.94 ± 0.03, HD 7.0 ± 4.5) performed reasonably well in case of segmentation of pathological hips from CT images. In comparison, the recent work of Wu et al. [15] reported DSC and HD of 0.936 ± 0.056 and 4.19 ± 1.04 mm for 282 pathological joints. However, on 30 healthy hips, the results reported by Wu et al. [15] were notably better (DSC 0.99 ± 0.014) than our results, like the work of Liu et al. [32] who reported similar metrics for hipbone segmentation on 221 metal-free CT images (DSC 0.99 and HD 3.30 mm). For comparison, we built an ensemble model composed of the fivefold models trained with Basic + SSIM augmentation and applied it on the 31 testing images of the subset “MSD_T 10” shared by Liu et al. in their CTPelvic1K public dataset [32]. This dataset provides some sort of external validation, although the corresponding CT images were only composed of hip bones without any particular bone pathology (femurs were not included as those were not segmented in the public dataset). We obtained an improved DSC of 0.95 ± 0.054 and an HD of 7.0 ± 5.8 mm but far from the performances of tailor-made models for CT (hip) bone segmentation [15, 18, 32]. As our THA training dataset contains several pathological cases with sometimes significant bone deformations and the presence of several implants, this distribution may have impacted the performance of the segmentation approach in the presence of healthy bones.

In conclusion, we proposed a novel augmentation varying both the shape and appearance of structures in generated images, which was successfully demonstrated with a deep learning approach to segment pathological hip bones from CT images.

As future work, it would be interesting to build other SSIM models from segmented public datasets and assess the impact of a SSIM-based augmentation in segmentation or classification tasks. It would be also valuable to compare our SSIM-based augmentation to other augmentation techniques involving both spatial and intensity augmentation such as GAN models.

## Data Availability

The datasets generated and/or analyzed during the current study are not publicly available due to restrictions imposed by the ethics committee.

## Abbreviations

DSC: Dice similarity coefficient
GAN: Generative adversarial network
HD: Hausdorff distance
PCA: Principal component analysis
SIM: Statistical intensity model
SSIM: Statistical shape and intensity model
SSM: Statistical shape model
THA: Total hip arthroplasty
TPS: Thin-plate spline

## Footnotes

1 While some authors use the umbrella term “SSM” to include models considering shape and/or appearance, we prefer to clearly distinguish models using SSM, SIM, and SSIM acronyms for shape, intensity, and hybrid models.
